# Advances in Drug Discovery Targeting Lysosomal Membrane Proteins

**DOI:** 10.3390/ph16040601

**Published:** 2023-04-17

**Authors:** Hongna Wang, Yidong Zhu, Huiyan Liu, Tianxiang Liang, Yongjie Wei

**Affiliations:** 1 Affiliated Cancer Hospital, Institute of Guangzhou Medical University, Guangzhou 510095, China; 2 Key Laboratory for Cell Homeostasis, Cancer Research of Guangdong Higher Education Institutes, Guangzhou 510095, China; 3 State Key Laboratory of Respiratory Disease, National Clinical Research Center for Respiratory Disease, Guangzhou Institute of Respiratory Health, Guangzhou 510095, China

**Keywords:** lysosome, drug, lysosomal membrane protein, therapy, disease, target

## Abstract

Lysosomes are essential organelles of eukaryotic cells and are responsible for various cellular functions, including endocytic degradation, extracellular secretion, and signal transduction. There are dozens of proteins localized to the lysosomal membrane that control the transport of ions and substances across the membrane and are integral to lysosomal function. Mutations or aberrant expression of these proteins trigger a variety of disorders, making them attractive targets for drug development for lysosomal disorder-related diseases. However, breakthroughs in R&D still await a deeper understanding of the underlying mechanisms and processes of how abnormalities in these membrane proteins induce related diseases. In this article, we summarize the current progress, challenges, and prospects for developing therapeutics targeting lysosomal membrane proteins for the treatment of lysosomal-associated diseases.

## 1. Introduction

Lysosomes are monolayer membrane-bound organelles found only in eukaryotic cells. They contain more than 60 hydrolytic enzymes that degrade a range of biomolecules and damaged organelles delivered to them by endocytosis and autophagy, and transport the degradation products to the cytoplasm or outside the cell for reuse. Thus, lysosomes have long been considered hydrolytic enzyme reservoirs and “recycling stations” for cells [1]. However, recent genomic, transcriptomic, proteomic, and bioinformatics studies have revealed that the lysosome is not only a terminal metabolic station of the cell, but is also involved in cell signaling, nutrient sensing, death, differentiation, secretion, and the quality control of proteins and organelles [2,3]. Therefore, lysosomal dysfunction can lead to impaired intracellular clearance of toxic substances, apoptosis, and abnormal cell signaling, resulting in various diseases. The most representative one is lysosomal storage disorders (LSDs), a rare metabolic disease caused by a genetic deficiency of specific lysosomal enzymes. Patients with LSDs experience the progressive deposition of undegraded metabolites in the lysosomes due to lysosomal hydrolase defects, causing cytopathy and dysfunction of various tissues and organs throughout the body, often leading to premature death [4].

Furthermore, neurodegenerative and cardiovascular diseases have been linked to decreased lysosomal function with age, and tumor cells have been found to upregulate lysosomal activity or biogenesis to meet the metabolic demands of their exuberant growth and proliferation [5,6,7]. Currently, cases of various other diseases caused by lysosomal dysfunction are rapidly accumulating, and as a result, the development of drugs and therapeutics targeting lysosomes is also on the rise. Since lysosomal membrane proteins are the primary regulators of lysosomal function, targeting them is also a current focus of drug development.

## 2. Functions of Lysosomes

In 1949, Christian de Duve, a biologist from Brussels, Belgium, set out to investigate how glucose-6-phosphatase responds to insulin regulation by determining its localization in the cell. He isolated various organelles from rat liver homogenates and was surprised to find glucose-6-phosphatase distributed on a new, previously unidentified organelle [8]. De Duve, in collaboration with Novikof, observed this organelle with electron microscopy for the first time in 1955 and then named it lysosome. In 1974, De Duve received the Nobel Prize in Physiology or Medicine for discovering lysosomes.

Lysosomes are formed by the fusion of transport vesicles carrying acidic hydrolases budded from the trans-Golgi network with endosomes, which contain molecules taken up by endocytosis at the plasma membrane. Lysosomes are the cell’s reservoir of hydrolases, and their lumen has more than 60 hydrolases that are active only at an acidic pH. Depending on the substrate degraded, lysosomal hydrolases can be classified into several species, including sulfatases, glycosidases, peptidases, phosphatases, lipases, and nucleases, which ensure that lysosomes efficiently degrade a broad range of substances delivered to them, such as mucopolysaccharides, sphingolipids, glycogen, and proteins [9]. The membrane-bound vacuolar ATPase (V-ATPase) continuously pumps H^+^ into the lysosomal lumen by hydrolyzing ATP to maintain its internal pH between 4.5 and 5.5. This acidic environment is critical for the lysosome to maintain its structural integrity and functions of hydrolase activation, calcium storage, vesicle transport, nutrient sensing, and signal transduction. In addition to V-ATPase, lysosomes contain approximately 50 membrane proteins, the most abundant of which are LAMP (lysosomal-associated membrane protein)1, LAMP2, LIMP (lysosomal integral membrane protein)-2, and cluster of differentiation (CD63) [10]. The intraluminal portions of these membrane proteins are highly glycosylated, forming a glycocalyx that protects the lysosomal membrane from digestion by acidic intraluminal hydrolases.

### 2.1. Lysosomal Degradation

**Figure 1:**
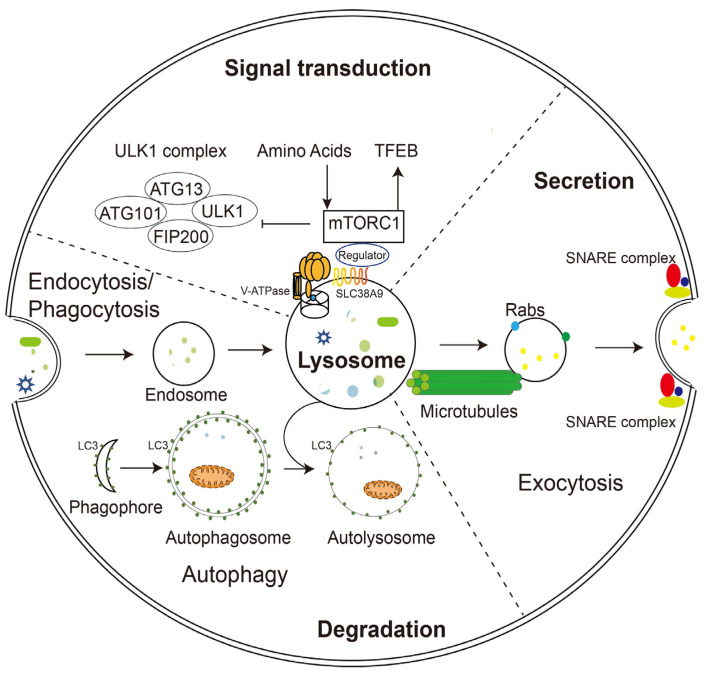
Schematic illustration of lysosomal functions. Lysosomes play a key role in endocytosis/phagocytosis, degradation, exocytosis, and signaling. Nutrients or foreign substances absorbed by endocytosis/phagocytosis enter the cytoplasm through the endosomal/lysosomal pathway. V-ATPase and SLC38A9 on the lysosomal membrane regulate nutrient signaling, TFEB, and autophagy mechanisms by interacting with mTORC1. During phagocytosis, lysosomes move along microtubules and fuse with the plasma membrane with the help of Rabs and SNARE complexes.

The unique composition and structure of lysosomes allow them to carry out various cellular functions such as degradation, secretion, and signaling. Unlike proteasomes, which degrade only proteins, lysosomes digest multiple substances, including proteins, glycosaminoglycans, nucleic acids, oligosaccharides, and complex lipids. The degradation substrates can be delivered to the lysosome through endocytosis, phagocytosis, or autophagy (Figure 1) [11]. Phagocytosis is restricted to specific mammalian cells called phagocytes (usually immune cells), whose function is to remove large pathogens such as bacteria and viruses, dead cell debris, and large dust particles. Endocytosis, which absorbs fluid and solute, on the other hand, occurs in all cells. Endocytosis/phagocytosis absorbs extracellular or surface cargos by plasma membrane-derived endocytic vesicles, which develop over time into early and late endosomes. Mature late endosomes fuse with lysosomes to form a hybrid structure called endolysosome, which carries out the bulk of degradation [12].

In parallel, harmful substances inside the cell, such as aggregated proteins and degenerated organelles, are sent to the lysosome for removal by autophagy (Figure 1). Depending on the mechanism by which degradation substrates are delivered to the lysosome, mammalian autophagy can be divided into three subtypes: in microautophagy, the lysosome engulfs cytoplasmic material through direct membrane invagination; chaperone-mediated autophagy (CMA) uses molecular chaperones carrying cargos to cross the lysosomal membrane directly without membrane remodeling with the assistance of the lysosomal protein LAMP2A; macroautophagy (the most well-studied and often referred to as autophagy) utilizes a particular double-membrane vesicle called autophagosome to encapsulate the cargo, and the autophagosomes eventually fuse with lysosomes to form autolysosomes where the bulk degradation occurs [13,14,15].

### 2.2. Lysosomal Exocytosis

In addition to degradation, lysosomes can also extracellularly release their contents in the form of lysosomal exocytosis (Figure 1). During this process, lysosomes migrate along microtubules from the perinuclear region toward the vicinity of the plasma membrane. They then dock and fuse directly with the plasma membrane through mechanisms involving the trans-SNARE complex formation and local Ca^2+^ release, and offload their contents into the extracellular compartment. Soluble *N*-ethylmaleimide-sensitive factor attachment protein receptors (SNAREs) are a group of membrane-associated proteins that play a key role in vesicle trafficking and fusion in eukaryotic cells. When a vesicle approaches its target membrane, the SNARE proteins on the vesicle (v-SNAREs) interact with complementary SNARE proteins on the target membrane (t-SNAREs), forming a stable complex that brings the two membranes together. The trans-SNARE complex is formed when v-SNAREs and t-SNAREs interact across two different membranes, leading to membrane fusion and the release of vesicle contents into the target compartment [16]. During fusion with the plasma membrane, some lysosomal membrane elements were retained and became a component of the plasma membrane, suggesting the potential of lysosomal exocytosis to remodel the cell membrane [1,17].

Lysosomal exocytosis is associated with two essential cellular functions, namely membrane repair/remodeling and secretion. Damage to the plasma membrane induces a rapid translocation of lysosomes to the damaged zone. After the local release of Ca^2+^, lysosomes undergo a series of events that culminate in the fusion of lysosomal membranes with the damaged plasma membrane. This fusion allows for the insertion of lysosomal membrane components into the damaged area, effectively repairing the plasma membrane. At the same time, lysosomal enzymes secreted extracellularly by lysosomes also promote endocytosis of the damaged membrane fragments, thus completing the repair process. Membrane repair occurs in all cell types, while membrane remodeling only occurs in specialized cells. It performs specific functions, for example, the cell membrane extension when macrophages engulf pathogens and the rapid elongation of the apical plasma membrane when neurites outgrow [18].

The secretion of lysosomal contents has also been indicated to be involved in different functions, most of which are related to the remodeling of the extracellular matrix by hydrolases released by lysosomal exocytosis. Although there is some evidence that these functions are common to all cell types, early studies overwhelmingly point to them being cell type specific, examples include the degranulation of cytotoxic T-lymphocytes [19], bone resorption by osteoclasts [20], parasite defense by eosinophils and mast cells [21,22], melanocyte pigmentation [23], platelet coagulation, and the release of sperm hydrolase during fertilization [24].

### 2.3. Signaling Function of Lysosomes

In addition to its long-recognized function as a recycling station for nutrient production, lysosome has been found to sense intracellular energy and extracellular nutrient status directly and to serve as a signaling hub for coordinating cellular catabolic and anabolic reactions (Figure 1). The lysosomal membrane provides channels (such as V-ATPase and SLC38A9) for nutrients and energy to regulate the mammalian target of rapamycin complex 1 (mTORC1) and the transcriptional factor EB (TFEB), two of the most crucial nutrient-sensitive protein complexes, as well as a dynamic platform for their assembly and activation [3,25].

mTORC1 is a master regulator of cell growth and metabolism and is activated only in the presence of growth factors and nutrients. When nutrients are adequate, mTOR is recruited to the lysosomal membrane, and kinase activity is initiated. Conversely, when nutrients are lacking, mTOR is inactivated and released from the lysosomal surface. TFEB, one of the best-known downstream effectors of mTORC1, is recruited to the lysosomal membrane and phosphorylated by active mTORC1 under nutrient-rich conditions, thus preventing it from entering the nucleus. When nutrient deficient and mTOR inactivated, TFEB is dephosphorylated and migrates to the nucleus, initiating the transcription of downstream genes that encodes lysosomal hydrolases and lysosomal membrane proteins. The high expression of these genes is fundamental for lysosomal biogenesis and autophagy [26,27].

TFEB binds directly to a specific E-box-like palindrome sequence called the coordinated lysosomal expression and regulation (CLEAR) motif, found in the promoter regions of most lysosomal genes and many autophagy genes. Therefore, when activated, it coordinates the expression of a wide range of proteins involved in lysosomal biogenesis and function, autophagy, and lysosomal exocytosis. Importantly, TFEB does not regulate the basal transcripts of its targets, but rather enhances their transcript levels in response to environmental cues such as nutrient depletion or stress. Overexpression of TFEB substantially increases the autophagic degradation of substrates such as long-lived proteins, lipid droplets, and damaged mitochondria, suggesting that this transcription factor has regulatory effects on both non-selective and selective autophagy [28].

Recent studies have linked the pathogenesis of multiple LSDs and the late-onset of neurodegenerative diseases to impaired autophagy and the accumulation of substrates that fail to degrade. Interestingly, autophagy defects in these pathological processes are often caused by the dysregulation of TFEB. TFEB has been shown to be involved in the clearance of protein aggregates, including β-amyloid and alpha-synuclein, which are hallmark features of neurodegenerative diseases such as Alzheimer’s disease, Parkinson’s disease, and Huntington’s disease. In addition, the overexpression of TFEB ameliorates the severity of disease phenotypes observed in several cellular and mouse models of lysosomal storage diseases (LSDs), including but not limited to polysulfatase deficiency, Batten disease, Pompe disease, Gaucher disease, and cystinosis. This improvement occurs through enhanced lysosomal function and the autophagic clearance of the accumulated substances [29]. These studies summarize the role of TFEB as a master regulator of lysosomal biogenesis and autophagy, and its pivotal function in the maintenance of cellular homeostasis and disease pathogenesis.

### 2.4. Restoration of Lysosomal Function

As the degradation center of the cell, the lysosome must maintain structural integrity to avoid the devastating consequences of leakage of the dozens of hydrolytic enzymes it houses. Under normal conditions, glycosylation of lysosomal membrane proteins is sufficient to maintain membrane stability against damage by luminal proteolytic enzymes. However, after continuous exposure to oxygen radicals, optical damage, and other irritations, the lysosomal membrane loses its integrity, leading to increased permeability and release of hydrolytic enzymes into the cytoplasm. Without timely repair, sustained lysosomal rupture may lead to the massive release of lysosomal contents, extensive acidification of the cytoplasm and cascade hydrolysis of the contents, and irreversible cellular damage. Early studies showed that moderate lysosomal damage could induce apoptosis, whereas extensive damage led to irreversible necrosis of a large number of cells. Subsequent evidence suggests that sustained lysosomal damage is closely associated with the development of almost all modes of cell death, including apoptosis, necrosis, pyroptosis, and ferroptosis. The specific type of death correlates with the type of cell and the degree of lysosomal damage [30].

To maintain their well-being and homeostasis, cells have developed a range of quality control mechanisms for damaged lysosomes, including repair, elimination, and regeneration. The endosomal sorting transport complex (ESCRT), a conserved transport system commonly found in eukaryotic cells, plays a key role in repairing damaged lysosomal membranes. ESCRT relies on its components apoptosis-associated gene-2-interacting protein X (ALIX) and tumor susceptibility gene 101 (TSG101) to tether the lysosomal membrane, and Ca^2+^ efflux from the ruptured lysosomes enhances the tethering efficiency [31]. Exactly how ESCRT repairs lysosomal membranes is unknown, but it may do so by inducing the formation of filamentous helices on the membrane surface and the contraction of the lipid bilayer.

When the lysosomal membrane is damaged beyond repair, it is eliminated by activating a selective form of autophagy called lysophagy. Proteins of the galectin (Gals) family play an important role in lysophagy. Gal3 senses the rupture and binds to the glycoproteins in the lysosomal membrane. After aggregation at the damage site, Gal3 is ubiquitinated and recruits preexisting phagophores for autophagy by binding P62 and LC3. It also recruits autophagy regulatory proteins such as uncoordinated-51-like kinase 1 (ULK1), Beclin 1, and autophagy-related protein 16L1 (ATG16L1) to de novo synthesize phagophore and further amplify lysophagy. Gal8 and Gal9 can also mediate lysophagy, but their mechanism of action is slightly different from that of Gal3 [32,33,34].

With the removal of damaged lysosomes by lysophagy and the reduction in the overall cellular lysosomal population, TFEB is activated to initiate the lysosomal regeneration. The process of lysosome biogenesis has been discussed in the previous section and will not be repeated here.

## 3. Lysosomal Membrane Proteins and Their Relationship with Diseases

The lysosomal membrane is a protective barrier for lysosomal integrity and a conduit for substances’ entry and exit. The membrane proteins of lysosomes are highly glycosylated and protect the membrane from the highly acidic internal environment and more than 60 hydrolases in the lumen. For example, LAMP1 and LAMP2, the two most abundant lysosomal membrane proteins, both contain more than ten glycosylation sites [35]. In addition, the import of hydrolases for lysosomal biogenesis and substrates to be degraded by lysosomes and the export of degradation products for reuse require the involvement of lysosomal membrane proteins. About twenty membrane proteins on lysosomes are metabolite transport channels, including calcium channels, potassium channels, cystine carriers, sialic acid transport proteins, and Niemann–Pick C1 (NPC1) proteins. They play essential roles in acidifying the lysosomal lumen, mediating cytoplasmic protein translocation, and transporting degradation products to the cytoplasm [10]. Accordingly, their abnormalities cause various diseases associated with lysosomal dysfunction [36]. In the following, we describe some disorders related to representative membrane protein dysfunction and the corresponding research advances in diagnosis and treatment (Table 1).

### 3.1. The Vacuolar-Type ATPase

**Figure 2:**
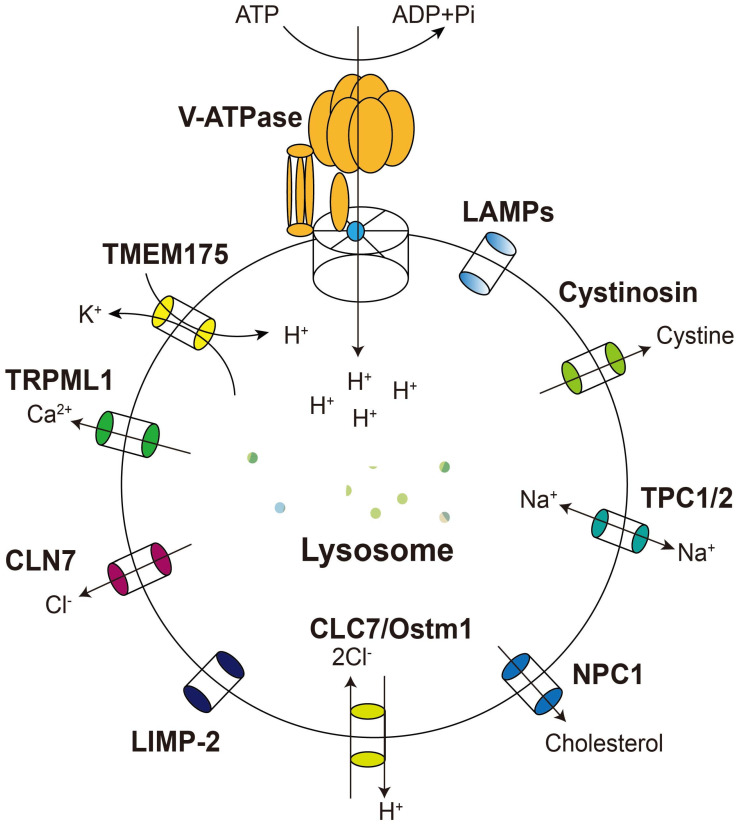
Lysosomal integral membrane proteins and their functions. The lysosomal integral membrane proteins discussed in this paper include LAMPs, LIMP-2, and various ion channels and transporters. LAMPs and LIMP-2 have been revealed to play a role in diseases such as cancer. The cation channels V–ATPase, TRPML1, TMEM175, and TPCs work together to regulate the concentrations of hydrogen, calcium, potassium, and sodium ions in lysosomes. The anion channels CLC7 and CLN7 are responsible for maintaining chloride ion balance. These ion channels help maintain the lysosomal membrane potential at −40 and −20 mV, which is necessary for lysosomes to perform their functions. The transporter proteins NPC1 and Cystinosin transport cholesterol and cystine, respectively.

The V-ATPase is an ATP-dependent proton pump widely distributed in the plasma membrane and many organelles such as lysosomes, endosomes, and secretory vesicles (Figure 2). It contains two structurally connected domains, V0 and V1. V0 is the transmembrane portion of V-ATPase, consisting of six subunits identified by lowercase letters a–e, in charge of proton transport; V1 is located in the cytoplasm, composed of eight subunits identified by capital letters A–H, responsible for ATP hydrolysis. Several subunits of mammalian V-ATPase are tissue-specific and are further identified by B1, B2, a1, a2, a3, etc. [58]. The primary function of V-ATPase is to actively pump H^+^ into the organelle or out of the cell using energy from ATP hydrolysis to create a proton gradient across the membranes. V-ATPase at the plasma membrane has received more attention and studies, but is not our focus in this article. It is involved in physiological processes such as bone resorption, sperm maturation, and urinary acidification, as well as pathological processes such as pathogen entry and cancer metastasis. Its dysfunction is associated with diseases such as osteoporosis and renal tubular acidosis [38,59].

The lysosomal V-ATPase continuously pumps H^+^ into the lumen and is essential for maintaining the acidic internal environment, hydrolase activity, and function of the lysosome. At the same time, the complexity of the V-ATPase composition means that its operation has a high chance of being compromised in various disease states, leading to abnormal acidification of the lysosome and accumulation of endocytic or autophagic cargos. The main pathological feature of many common neurodegenerative diseases, such as Alzheimer’s disease (AD), Parkinson’s disease (PD), Huntington’s disease, and amyotrophic lateral sclerosis (ALS), is the accumulation of misfolded protein aggregates caused by the inappropriate acidification of lysosomes [7]. Therefore, it has long been hypothesized that lysosomal V-ATPase dysfunction is a major causative factor in neurodegenerative diseases. This idea is supported by the clinical observation that some familial neurodegenerative diseases are caused by mutations in V-ATPase subunits or accessory proteins (e.g., ATP6AP2), and in vitro experiments that loss-of-function mutations in V-ATPase lead to impaired proteolysis and age-related neurodegenerative disease in Drosophila [60,61].

Many neurodegenerative diseases are not directly caused by defective V-ATPase mutations but exhibit abnormal lysosomal acidification and dysfunction. For example, loss-of-function mutations in presenilin-1/2 (PS1/PS2), ubiquitin-2/4 (UBQLN2/UBQLN4), or leucine-rich repeat kinase 2 are the leading causes of familial AD, ALS, and PD, respectively. Mechanistically, all these proteins bind directly to the V-ATPase a1 subunit [62,63,64]. In contrast, the pathogenic mutants lose this interaction, resulting in improper assembly and loss of function of the V-ATPase complex at the lysosomal membrane. Therefore, pharmacological manipulation of V-ATPase activity to restore proper acidity of lysosomes seems attractive for treating these diseases. For example, FK506 exerts neuroprotective effects by inducing autophagy through binding to ATP6V1A [41]. Dendrobium alkaloids (DNLA) increase the A1 subunit of V-ATPase levels in APP/PS1 mice and improve learning and memory function in this AD model [65].

Some diseases, most notably tumors, rely on autophagy to survive and progress. Subunits of V-ATPase are frequently overexpressed in tumor cells to accommodate autophagic demands, and thus the cells are more sensitive to V-ATPase inhibition [66]. Therefore, inhibition of V-ATPase activity becomes an attractive strategy for treating cancers. The V-ATPase inhibitors that have long been identified, such as bafilomycin A1, concanamycin A, Archazolid A, and INDO L0, are broad-spectrum and have no cellular or organelle specificity [67]. Bafilomycin A1 and concanamycin A inhibit V-ATPase in all eukaryotes at nanomolar concentrations and trigger programmed cell death in many cells at concentrations above 25 nmol/L [68]. Great efforts have been made to develop more potent and selective V-ATPase inhibitors suitable for clinical use by the functional screening of natural and synthetic bafilomycin A derivatives. However, the screening has mainly focused on cell specificity, and no progress has been made in the selectivity for lysosomal V-ATPase. SB242784 is 1000-fold more selective for osteoclasts than cells from the kidney, liver, spleen, stomach, and other tissues. Its administration successfully prevented the loss of bone components in osteoporotic rats [69]. Salicylihalamide A and its derivative saliphenylhalamide (saliPhe), Archazolid, and omeprazole of the indole family all exhibit good anticancer activity against different cancer cells (Table 1). Still, they act mainly by inhibiting V-ATPase on the plasma membrane [67]. The small molecule indole derivative NiK12192 indiscriminately reduces the volume and acidity of lysosomes in cancer and non-cancer cells and thus may have unpredictable side effects [70].

### 3.2. Lysosomal Calcium Channel TRPML1

TRPML1/MCOLN1 is a calcium channel in the lysosomal membrane and a member of the TRP (transient receptor potential) channel superfamily widely expressed in many different tissues and cell types (Figure 2) [71]. Most TRPs are non-selective cation channels, with only a few being Ca^2+^-selective, and they serve as gatekeepers for the transmembrane transport of a wide range of cations [72]. TRPML1 was first identified in mucolipidosis type IV (MLIV) patients, an autosomal recessive lysosomal storage disorder that often occurs in children. Mutations in the TRPML1-coding gene *MCOLIN1* are solely responsible for MILV. Patients with MLIV suffer from severe neurological and ophthalmologic abnormalities [73]. At the cellular level, fibroblasts from MLIV patients show enlarged endo-lysosomes, impaired autophagy, and accumulation of lipids and glycosaminoglycans [71]. It has been suggested that TRMPL1 may be involved in CMA, helping the CMA receptor lysosomal-associated membrane protein 2A (LAMP-2A) to transport substrates across the lysosomal membrane into the lumen. Thus, TRPML1 deficiency leads to impaired CMA and accumulation of damaged proteins and organelles, and induces MILV [74]. Later studies with knockout mice also showed that, in addition to MILV, abnormalities in TRPML1 are associated with other disorders such as Alzheimer’s disease (AD), Parkinson’s disease (PD), Niemann–Pick type C disease (NPC), and amyotrophic lateral sclerosis (ALS) [75,76]. Although the mechanism by which TRPML1 causes these diseases remains unclear, the evidence mainly points to an association with abnormal lysosomal Ca^2+^ efflux accompanied by lysosomal dysfunction, and modulation of TRPML1 function with activators or inhibitors appears to be a promising strategy for their treatment.

Human TRPML1 is a 65 kDa protein with 580 residues and is highly expressed in the heart, brain, kidney, spleen, and liver. TRPML1 is localized on the membrane of lysosomes and transports Ca^2+^ out of the membrane, which is essential for maintaining Ca^2+^ homeostasis and Ca^2+^ signaling in the lysosome [77]. Deficiency or dysfunction of TRPML1 leads to impaired lysosomal function and abnormal accumulation of heterogeneous substances in the lysosome [78]. Recent studies have identified endogenous and synthetic compounds that act on TRPML1 to study the role of TRPML1 and treat lysosomal storage disorders. Two native phosphatidylinositol isomers with similar structures, namely phosphatidylinositol 4,5-bisphosphate (PI(4,5)P2) and phosphatidylinositol 3,5-bisphosphate (PI(3,5)P2), regulate TRPML1 channels with opposite results. PI(3,5)P2 presents predominantly on late endosomes/lysosomes and promotes TRPML1 opening at a low pH; in contrast, PI(4,5)P2, primarily distributed on the cytoplasmic membrane, causes TRPML1 to close [79,80]. Depletion of PI(3,5)P2 in primary neurons with YM201636, a selective PIKfyve inhibitor, produced endo-lysosomal neuropathological features of AD, such as lysosomal swelling, accumulation of autophagic vacuoles (AVs), and elevated endo-lysosomal Ca^2+^ levels [81]. In contrast, reactivation of TRPML1 with the synthetic agonist ML-SA1 reversed the pathological changes brought about by YM201636 and restored the normal endo-lysosomal morphology and function of neurons [44]. ML-SA1 is more potent than PI(3,5)P2 and can operate alone or in synergy with PI(3,5)P2 to activate TRPML1 more effectively [82]. It has been used to study several disorders mentioned above resulting from impaired lysosomal function, such as MLIV, NPC, and AD. Other small molecule agonists of TRMPL1, such as SF-22 and MK6-83, have also been developed and successfully used to restore TRPML1 activity and rescue disease-associated abnormalities in fibroblasts from MLIV patients (Table 1) [83,84]. More importantly, in 2019, Merck acquired Calporta Therapeutics, the small molecule therapies developer that invented ML-SA1, for USD 576 million, which is a massive boost to using TRPML1 as a therapeutic target for LSDs.

### 3.3. Lysosomal Potassium Channel TMEM175

TMEM175 is a lysosomal membrane protein initially identified as a K^+^ efflux channel, but its potassium channel activity has only been tested in vitro at high pH conditions. Therefore, whether it is responsible for K^+^ efflux inside the acidic lysosomal lumen in vivo remains debatable. TMEM175 was recently found to be activated as an H^+^ channel at acidic pH (4.5–5.0) in the lysosome, where it is highly permeable and selective for protons compared to potassium. It drives a small amount of H^+^ leakage to balance the V-ATPase-mediated influx, thus maintaining the pH in the lysosomal lumen in the optimal range of 4.5–5.0 (Figure 2) [47]. TMEM175 defect leads to the excessive acidification of lysosomes and a reduced degradation capacity, dysfunctional autophagy, and accumulation of undegraded substrates in lysosomes [85].

Multiple genome-wide association studies (GWAS) have identified a high correlation between the p.M393T variant in TMEM175 and Parkinson’s disease [86,87]. Knockout of TMEM175 in mouse neurons leads to the hyper-acidification of lysosomes, impaired lysosomal hydrolytic activity, and aggregation of a-synuclein in the brain, a typical pathological feature of PD [88]. Overexpression of wild-type TMEM175 in rat primary hippocampal neurons reduced the p-α-syn inclusion. In contrast, the overexpression of p.M393T exerted a dominant-negative effect and partially recapitulated the TMEM175 knockout phenotype. Mechanistically, the M393T mutant has reduced function in both K^+^ and proton permeation compared to wild-type TMEM175 and therefore is deficient in maintaining lysosomal pH stability [89].

The endogenous metabolite arachidonic acid (ArA) can activate TMEM175 in a pH-independent manner and increase the K^+^ and H^+^ permeability of lysosomes, making them more sensitive to osmotic shock. Given that ArA is an integral component of biological cell membranes and is also involved in many types of cellular signaling, it is worthwhile to investigate whether ArA imbalance contributes to TMEM175 dysfunction and LSD. DCPIB and ML67-33, two synthetic small molecule drugs that alter other iron channels, were also found to activate TMEM175 and promote H^+^ leakage from lysosomes. Although it is yet to be investigated whether DCPIB and ML67-33 can promote the clearance of p-α-syn aggregates in PD neurons (especially those from PD patients carrying the M393T TMEM175 variant), activation of TMEM175 to restore lysosomal function appears to be an attractive therapeutic approach of PD (Table 1) [47,90]. The design and screening of more effective and selective TMEM175 agonists rely on a thorough understanding of the mechanism of TMEM175 activation and a detailed dissection of how it maintains lysosomal proton homeostasis and function.

### 3.4. CLC7

CLC7 is a member of the CLC protein family, which contains plasma membrane-localized Cl-channels (ClC-1, ClC-2, and ClC-Ks) and 2Cl^−^/H^+^ antiporters (CLC3 through CLC7) located on distinct but overlapping compartments of the endosomal–lysosomal pathway. It is the only lysosome-resident CLC and requires a beta subunit, the osteoporosis-associated transmembrane protein 1 (OSTM1), for its proper expression, localization, and transport activity (Figure 2) [91]. The heavily glycosylated, single-pass transmembrane protein Ostm1 binds strictly to the un-glycosylated CLC7, protecting it from degradation in the acidic, protease-rich lysosomal lumen [92]. In parallel, Ostm1 relies on CLC7 to leave the ER and be directed to the lysosome, where it is cleaved to reach maturation [93]. Thus, they are mutually dependent, and knockdown of one protein will inevitably destabilize the other [91].

CLC7/Ostm1 are ubiquitously expressed, with particularly high levels of expression in the central and peripheral nervous system, and they are predominantly localized to the lysosomes. In osteoclasts, they are also present, in addition to lysosomes, at the ruffled border, a specialized plasma membrane region sealed to the bone surface that defines the site of resorption of bone material by osteoclasts [94]. Loss-of-function mutations in CLC7 and Ostm1 results in osteopetrosis, lysosomal storage diseases, and neurodegeneration in both mice and humans. Results of subsequent experiments with various *Clcn-7* mutant mice showed that the phenotypes of neurodegenerative disease and osteoporosis are independent outcomes caused by defects in lysosomal- and ruffled border-localized CLC7, respectively, rather than secondary to each other. Evidence includes, but is not limited to: (1) *Clcn7^−/−^* mice that specifically and exogenously express CLC7 in osteoblasts and macrophages that do not have osteopetrosis but have severe retinal and CNS degeneration similar to whole knockout mice; (2) CLC7-targeted mice that express an alternative transcript only in bone exhibit neurodegeneration but no osteopetrosis [95].

Initially, CLC7 was thought to be a Cl^−^ channel that collaborates with v-ATPase while maintaining effective acidification of the lysosomal lumen. However, the evidence opposes this notion as the pH in the lysosomes of *Clcn7^−/−^* and *Ostm1^−/−^* mice is normal, while Cl^−^ does decrease. The perception of the role of CLC7 in lysosomal biology changed significantly after it was determined that CLC7 is not a Cl^−^ channel, but a coupled 2Cl^−^/1H^+^ antiporter. It was then proposed that the main role of CLC7 is to increase the Cl^−^ concentration in the lumen using the pH gradient generated by H^+^-ATPase. It is unclear why high Cl^−^ concentrations are important for lysosomal function, but it may be that some degradative enzymes, such as cathepsin C, and lysosomal Ca^2+^ channels, are directly regulated by Cl^−^ [96,97]. Echoing this, lysosomal chloride concentrations have recently been found to be reduced in several lysosomal storage diseases unrelated to primary defects in lysosomal Cl^−^ transport in both *Caenorhabditis elegans* (*C. elegans*) and mammalian cell models [97].

The phenotype of a *Clcn7* knock-in mice, *Clcn7* ^td/td^, suggests a new function of ClC-7/Ostm1 beyond its transport activity, although the mechanism remains to be further revealed. In *Clcn7* ^td/td^ mice, the ‘proton glutamate’ E312 of CLC7 was mutated to alanine, thereby abolishing both Cl^−^ and H^+^ transport by CLC7/Ostm1. These mice exhibited the same severe osteoporosis phenotype as the *Clcn7^−/−^* mice, but the fur color was not affected. Fur pigment in mice is synthesized in the melanosomes (compartments associated with lysosomes) of melanocytes, and gray fur is an indicator of melanocyte lysosomal disorder [98]. In addition, both loss-of-function and acquired mutations in CLC7 may be pathogenic, further suggesting that it functions beyond Cl^−^/H^+^ transport.

Given the association of clcn7 mutation with osteoporosis, CLC7 is often suggested as a target for the treatment of this disease. Chloride channel inhibitor NS3736 and its analogs successfully inhibited osteoclast resorption in vitro and prevented bone loss in vivo in a rat model of osteoporosis (Table 1) [48]. However, whether they act directly on CLC7, whether they also work on lysosomal CLC7, and whether they alleviate neurodegenerative symptoms remains to be tested. The recently solved cryo-EM structure of the CLC7/ostm1 complex will undoubtedly provide great insight into the development of drugs targeting CLC7.

### 3.5. NPC

NPC1 is a late endosomal/lysosomal membrane protein with 1278 amino acids and 13 transmembrane helices, while NPC2 is a lysosomal intraluminal protein with only one structural domain, which together participate in the trafficking of LDL-derived cholesterol (Figure 2) [99]. Cholesterol-rich low-density lipoprotein (LDL) is first endocytosed via the LDL receptor into the cell. Then, in the endosome or lysosome, the NPC2 protein strips cholesterol from LDL and transfers it to NPC1, where it is transported to other cell sites for further utilization. Therefore, mutations in NPC1 often lead to abnormal accumulation of cholesterol in lysosomes, causing an excessive build-up of fat lipids in the liver, kidneys, spleen, and even brain, eventually resulting in Niemann–Pick disease (NPC) [100]. Ninety-five percent of NPC patients diagnosed so far are due to mutations in the gene encoding NPC1, while the other 5% are attributed to mutations in NPC2. There is no effective cure for NPC, and symptomatic therapy is currently used to improve patients’ neurological function and quality of life [101,102].

Zavesca (Miglustat) is the only drug approved for treating NPC in Europe, Australia, and Japan, and its early usage reduces neurological symptoms and delays disease progression [103]. Recently, researchers have used AAV vectors to deliver Npc1-encoding cDNA into *Npc1^−/−^* mice or NPC1-encoding mRNA into fibroblasts derived from NPC patients, both of which rescued NPC symptoms in these mice or cells, suggesting that gene therapy may offer a glimpse of hope for NPC patients (Table 1) [49,104].

In addition to its role in cholesterol transportation, NPC has also been found to act as an intracellular receptor for the Ebola virus in its invasion process. A genome-wide haploid genetic screen identified NPC1 as one of the host factors for filovirus entry, and NPC1-deficient primary fibroblasts from NPC patients were resistant to the Ebola virus, further confirming the indispensability of NPC1 for Ebola infection [105]. Benzyl piperazine adamantane diamine-derived compounds inhibited Ebola virus replication, and NPC1 was identified as the target in further assays using mutant cell lines and informative derivatives of the lead compound (Table 1) [50]. Mechanistically, the glycoprotein (GP) on the surface of the Ebola virus is cleaved in the lysosome to cleaved-GP (GPcl), which in turn binds directly to NPC1 and initiates the membrane fusion of the virus with the host organelle [106]. A 6.6 Å resolution cryo-electron microscopic structure of the NPC1 and GPcl protein complex shows that the NPC1 protein monomer and the GPcl trimer recognize each other through a single interface, which is consistent with the previously resolved crystal structure of the complex formed by the NPC1 C-domain and GPcl [107,108]. Advances in structural studies provide a molecular basis for studying the mechanism of NPC1-mediated Ebola virus invasion and for designing antiviral drugs to interfere with this process by disrupting the NPC1-virus GPcl recognition interface.

### 3.6. TPCs

Two Pore Channels (TPCs)are a family of cation-selective ion channels with two sets of six-helical transmembrane domains. There are three TPC isomers in animals, TPC1, TPC2, and TPC3, and only TPC1 and TPC2 are expressed in primates or rodents. The distribution of TPCs in cells is varied, with TPC1 and TPC3 at the early and circulating endosomes and TPC2 at the late endosome and lysosomes (Figure 2) [109]. Whether TPC1 is a nicotinic acid adenine dinucleotide phosphate (NAADP)-activated Ca^2+^ influx channel, a PI(3,5)P2-activated Na^+^ channel, or both is controversial. However, its activity is usually considered to be regulated by voltage. On the other hand, TPC2 is voltage-insensitive and can be activated both by the lysosome-specific PI(3,5)P2 to conduct Na^+^ and by NAADP, PI(3,5)P2 or Mg^2+^ to efflux Ca^2+^ [110,111,112]. The recently resolved atomic structure of the NPC complex shows that PI(3,5)P2 binds directly to TPC2 and that mutations of complementary basic amino acids in the binding pocket abolish the activation by the phospholipids [113]. Meanwhile, NAADP indirectly stimulates TPC2 by binding to the accessory proteins LSm12 and JPT2 [114].

To date, most of the physiological functions of TPC2 have been related to the Ca^2+^ signaling and lysosomal processes it maintains. In neurons, the neurotransmitter glutamate uses NAADP to turn on Ca^2+^ signaling, which further drives autophagy through TPC2 to maintain neuronal homeostasis and function [115]. Defects in TPC2 may lead to neurodegeneration, resulting in AD and PD [116]. TPC2 has been reported to be involved in angiogenesis and embryonic myogenesis in the vascular system, and its abnormalities lead to cardiovascular complications [109].

Metabolically, TPC2-mediated Ca^2+^ efflux is involved in starvation-induced mTORC1 inactivation on the lysosomal surface. Reciprocally, mTOR phosphorylates and inhibits TPC2 in the presence of adequate nutrients. Thus, when TPC2 is knocked out, mTORC1 maintains high activity even under nutrient deficiency. Mice lacking TPC2 are more susceptible to fatty liver diseases from a high-fat diet due to reduced cholesterol/triglyceride clearance triggered by mTOR dysregulation [112].

There is growing evidence that TPC2 influences different aspects of cancer development. TPC2-mediated angiogenesis may provide the blood supply for tumor growth, while tumor proliferation, migration, and metastatic invasion have also been found to be controlled by TPC2. Consequently, genetic or pharmacological disruption of TPC2 leads to tumor regression, suggesting that TPC2 is an attractive target for cancer intervention [117,118].

Viruses often exploit the host’s endocytic system to invade. Therefore, TPC2 is often involved in the invasion and has the potential to be a target for antiviral drugs. Knockdown of TPCs or inhibition of TPCs with Ned-19, a selective membrane-permeable non-competitive NAADP antagonist, inhibited infection by viruses such as Ebola, MERS-COV, and SARS-CoV-2 and prevented them from attacking the host’s myocardial system (Table 1) [51,119].

### 3.7. CLN7

Batten diseases, also known as neuronal ceroid lipofuscinoses (NCLs), is a general term for a spectrum of inherited lysosomal storage disorders with similar clinical manifestations, first discovered by and named after the British neurologist Frederick Batten. The common pathological feature of NCLs is the presence of spontaneous deposits of fluorescent substances in the lysosomes and extensive neuronal death. Each subtype of NCLs is classified by the gene that causes it, and each gene begins with CLN (ceroid lipofuscinoses neuronal), followed by a unique number representing the subtype [120]. The CLN7 subtype of Batten disease is caused by a mutant in the *CLN7* gene, which disrupts the normal function of the lysosomal transmembrane protein it encodes. CLN7 patients present in early childhood with neurological symptoms such as seizures, progressive mental and motor abilities’ deterioration, and loss of vision, eventually leading to a shortened life span [121].

Since CLN7 has only recently been identified as a novel lysosomal chloride channel and its function was previously mostly unknown, specific therapies targeting CLN7 have not yet been developed [122]. Symptomatic treatment may provide some benefit, but not enough to stop disease progression or prevent premature death. In recent experimental gene therapy, the wild-type *cln7* gene was delivered with an AAV vector into a *cln7^−/−^* mouse model of CLN7 Batten disease (Table 1). Lower levels of CLN7 expression in central and peripheral nerves were sufficient to increase neuronal lysosomal activity and reduce lysosomal storage, resulting in reduced neuroinflammation, improved neurobehavior, and longer life span in mice. Higher expression of CLN7 does not provide additional benefits [123]. Many questions remain regarding the dosing and timing of AAV administration. However, this work is a substantial advance for a very challenging disease and paves the way for Phase I clinical trials.

### 3.8. LAMP1 and LAMP2

Lysosome-associated membrane proteins (LAMPs) are a group of integral membrane proteins found specifically in lysosomes (Figure 2) [10]. The LAMPs family includes five members, among which LAMP1 and LAMP2 are universally expressed in all cell lines and tissues, while LAMP3, LAMP4, and LAMP5 are cell-specific and will not be discussed here [124]. LAMP1 and LAMP2 constitute half of all lysosomal membrane proteins and help maintain lysosomal pH, integrity, and catabolism [125]. In LAMP1-knockout mice, an increase in LAMP2 expression was observed, but no significant phenotypic changes were reported [126]. As a result, determining the precise function of LAMP1 is difficult, but it appears that LAMP2 can compensate for the loss of LAMP1 function to some degree in vivo [126]. Recent research in *Drosophila* suggests that LAMP1 may have a neuroprotective effect by promoting the development of non-pathogenic aggregates in neurons, neutralizing the toxicity of α-synuclein. In a *Drosophila* model of Parkinson’s disease, LAMP1 deletion increases sensitivity to alpha-synuclein and oxidative stress [127]. In line with this, Cawley et al. found that abnormal LAMP1 glycosylation might contribute significantly to NPC progression, with high glycosylation levels of LAMP1 detected in both *Npc*^−/−^ mice and NPC patients [128]. LAMP2 plays a vital role in endosomal/lysosomal-mediated cholesterol exportation. It has three isoforms, LAMP2A, LAMP2B, and LAMP2C, and overexpression of any of these isoforms reduces cholesterol aggregation in late endosomes/lysosomes [129]. Mutations in the *LAMP2* gene can lead to Danon disease, an X-linked dominant disease in which patients have weakened bones and heart muscle, leading to multi-organ disease, severe heart failure, and ultimately death [36]. There is still no effective treatment for Danon disease, and organ transplantation is the only option for patients. A recent study using AAV9 to restore LAMP2 expression in a *lamp2* knockout mouse model of Danon disease yielded encouraging results. AAV-dependent LAMP2B protein expression was detected in the heart, liver, and skeletal muscle of mice that received gene therapy (Table 1). Mice in the treated group showed reduced hepatic transaminases, improved cardiac function, and significantly improved survival after receiving high doses of AAV-LAMP2 as adults [53]. These results suggest that *LAMP2B* gene therapy can potentially treat this severe genetic disease. The team is recruiting male volunteers with Danon disease to conduct clinical trials for gene therapy.

LAMP1 and LAMP2 expression has been identified on the surface of cancerous tumors, especially in highly metastatic cancers such as colon cancer and melanoma, implicating them in tumor cell metastasis [130,131]. However, their functions as non-lysosomal localized proteins are not within the scope of our discussion and will not be elaborated on here.

### 3.9. Cystinosin

Cysteine is the least abundant and often restricted intracellular amino acid and is usually produced by the reduction in cystine in the cytosol. Lysosomal cystine, a by-product of lysosomal protein hydrolysis, is the principal intracellular reservoir of cysteine, and its concentration is 30-fold higher than in the cytoplasm during cell proliferation. Cystine efflux is mediated by the proton-coupled transporter cystinosin, which regulates intracellular cysteine levels for several fundamental activities, such as glutathione synthesis and tRNA thiolation (Figure 2) [132]. Dysfunctional mutations in the *CTNS* gene encoding cystinosin cause cystine to accumulate in the lysosomes and form crystals in most tissues, leading to the life-threatening disease cystinosis. Cystinosis primarily affects the patient’s renal tubules and corneas in the early stages and then gradually extends to other organs [133].

The primary treatment for cystinosis is the lifelong use of the sulfhydryl drug cysteamine, which chemically reduces cystine to form mixed disulfides that can exit the lysosome via the alternative PQLC2 channel (Table 1) [55]. Although cysteamine therapy has dramatically improved patients’ quality of life with cystinosis, it is not a curative therapy as it does not restore functional cystinosin or cystinosin-mediated signals. A recent study has raised the possibility that patients with cystinosis carrying nonsense *CTNS* mutations, such as the w138x mutation often found in French Canadians, could be treated with drugs that stimulate translational readthrough. Aminoglycoside antibiotics, such as geneticin (G418), bind to mammalian ribosomes, reduce translation fidelity, and inhibit translation termination caused by premature termination codons (PTC). G418 successfully restores CTNS expression and reduces pathological cystine accumulation in fibroblasts from patients carrying the W138X mutation. ELX-02 is a fifth-generation aminoglycoside designed by Eloxx Pharmaceuticals that possesses a readthrough effect of PTC comparable to G418 but without significant toxic effects (Table 1). It effectively reduced cystine accumulation in the kidneys of mice carrying the *CTNS Y226X* mutation (another PTC mutation) without causing cytotoxicity or nephrotoxicity [134]. These results demonstrate the potential of ELX-02 for the treatment of cystinosis, and the relevant Phase I and Phase II clinical trials are currently underway.

Cystinosins belong to the PQ-loop transporter protein family, which possesses two conserved proline-glutamine dipeptide repeats known as PQ-motifs. It has seven transmembrane helices and drives cystine efflux from the lysosome in a 1:1 ratio using an electrochemical gradient of outgoing proton produced by V-ATPase. Selective targeting of cystinosin to alleviate the crippling cystine transport that underlies cystinosis holds the promise of a cure for this disease. Progress in this area depends on the complete revelation of the mechanistic link between cystinosis symptoms and *CTNS* gene mutations at the molecular level [135]. Two recent structural studies of cystinosin open the door to manipulating its cystine transporting activity. Guo et al. solved the cryo-EM structures of human cystinosin in the lumen-open, cytosol-open, and cystine-bound states, revealing the mechanism of cystine recognition and capturing the critical conformational states of the transport cycle [132]. Löbel et al. revealed the crystal structure of cystinosin from *Arabidopsis thaliana* in apo and cystine-bound states and established a mechanism for cystine recognition and proton-coupled transport [135]. The structural and functional data presented in these two studies, combined with the functional annotation of key pathogenic mutations, provide a solid basis for developing a molecular blueprint for lysosomal cystine transport and cystinosis.

### 3.10. LIMP-2

Lysosomal integral membrane protein 2 (LIMP-2) is a sorting receptor for mammalian β-glucosidase (GCase) and is responsible for transporting GCase from the endoplasmic reticulum (ER) to the lysosome via endolysosomal compartments. It binds and loads GCase at the neutral pH of the ER and unloads GCase at the acidic pH of the lysosome. Loss-of-function mutations in the gene encoding GCase, *glucosyl ceramidase beta 1* (*GBA1*), lead to Gaucher’s disease (GD), one of the most representative LSDs [136]. LIMP-2 deficiency usually leads to a severe reduction in GCase activity in various tissues. Thus, mutations in *SCARB2* (the gene encoding LIMP-2) often lead to a certain phenotypic spectrum of GD, such as a rare form of progressive myoclonic epilepsy (PME) often associated with action myoclonus-renal failure syndrome (AMRF) [137]. Unlike other chaperones that cycle between the Golgi and lysosomes, LIMP-2 resides primarily in the lysosome, suggesting that it has other functions besides being a lysosomal enzyme receptor. A recent study identified LIMP-2 as a novel lysosomal lipid transporter that transports cholesterol (and may be other lipids) to the lysosomal membrane with the cavity in its luminal domain. It operates in parallel with the Niemann–Pick (NPC) proteins, but in a slower mode, mediating the export of lysosomal cholesterol [138]. Both LIMP-2 functions in lysosomal GCase import and lipid export may contribute to lipid storage and autophagy-lysosomal dysfunction in LIMP-2-deficient mice, resulting in α-synuclein accumulation and neuronal toxicity. Overexpression of LIMP-2 in murine neuroblastoma as well as human glioma cells accelerated the clearance of α-synuclein, demonstrating the potential of exogenous expression of LIMP-2 in GD gene therapy (Table 1) [57].

## 4. Conclusions

Lysosomes are the predominant organelles for the degradation of macromolecules in eukaryotic cells. Recent studies have revealed that in addition to degradation, lysosomes are also involved in various physiological processes such as autophagy, nutrient sensing, and intracellular signaling. As the link between lysosomal abnormalities and an increasing number of diseases, especially neurodegenerative disorders, has been revealed, targeting lysosomes has gradually become an essential direction for drug development. In recent years, various lysosomal ion channels such as the TRPML1, TPC2, and TMEM175 have been identified, providing a starting point for understanding the mechanisms by which lysosomal abnormalities cause diseases and targeting them for the treatment of these diseases. Several lysosomal functions modulating small molecule compounds targeting lysosomal membrane proteins, including V-ATPase inhibitors and ion channel modulators, have been developed or are in development and have shown promising therapeutic potential. This article briefly reviews the current progress of research and drug development on lysosomal membrane proteins, hoping to shed some light on treating diseases related to lysosomal abnormalities.

## Figures and Tables

**Table 1:** Modulators/therapies targeting lysosomal membrane proteins and related preclinical/clinical studies.

Target	Functions	Chemicals/Therapies	Mechanism	Stage of Development	Disease/Potential Application
V-ATPase	Acidification of lysosome	Bafilomycin A1, Concanamycin A, INDO L0	Inhibit c subunit of V0 domain in V-ATPase	Tool compound/Preclinical	Cancer [37]
		SB242784	With high potency and selectivity for the c, a, or V0 domain of osteoclast V-ATPase	Preclinical	Osteoporosis [38]
		Salicylihalamide A, SaliPhe, Archazolid	Inhibit V0 domain of V-ATPase	Preclinical	Cancer [39,40]
		FK506	Protect nervous system through binding with ATP6V1A and inducing autophagy	Preclinical	Neuronal cells [41]
		NiK12192	Indole derivative that causes a reduction in the volume and acidity of lysosomes, leads to antimetastatic effect	Preclinical	Cancer [42]
TRPML1	Regulate lysosomal calcium concentration	ML-SA1	TRPML1 activator	Preclinical	Neurodegenerative diseases, MLIV [43,44,45]
		PI (4,5) P2	TRPML1 activator		
		PI (3,5) P2	TRPML1 agonists		
		SF-22	TRPML1 activator		
		MK6-83	TRPML1 activator		
TMEM175	Modulate lysosomal potassium and proton	4-AP	Inhibit the K^+^ channel activity of TMEM175 [46]	Phase III	Spinal muscular atrophy (NCT01645787), Guillain-Barre Syndrome (NCT00056810)
		ArA	Activate TMEM175 and enhance the permeability of K^+^ and H^+^	Preclinical	Neurodegenerative disease [47]
		DCPIB	Ion channel activator		
		ML67-33	Ion channel activator		
CLC7	Lysosomal chloride channel	NS3736	Chloride channel inhibitor	Preclinical	Osteoporosis [48]
NPC1	Lysosomal cholesterol channel, intracellular receptor for Ebola virus	NPC1-AAV9 gene therapy	Correct NPC1 mutation and restore the function of NPC1 [49]	Clinical	Niemann–Pick disease, type C [49]
		Benzylpiperazine adamantane diamide 3.0	Inhibit NPC1 to block viral entry	Preclinical	Ebola virus infection [50]
TPCs	Na^+^ channel	Ned19	NAADP antagonist, block TPC	Preclinical	Viral infection [51]
		NAADP	Activate TPC		
		PI (3,5) P2	Activate TPC2		
CLN7	Lysosomal chloride channel	Gene therapy	Rescue CLN7	Clinical	Batten Disease [52]
LAMP2	Lysosomal membrane structural protein	LAMP2B-AAV9 gene therapy	Restore the function of LAMP2B [53]	Phase I	Danon Disease (NCT03882437)
Cystinosin	Transport cystine	ELX-02	Binds to ribosomes and rescue cystinosin W138X mutation [54]	Phase II	Cystinosis (NCT04069260)
		Cysteamine	Binds to lysosomal cystine and transport them out through lysosomal cationic amino acid transporter	FDA-approved drug	Cystinosis [55,56]
LIMP-2	Lysosomal membrane structural protein	Gene therapy	Restore the function of LIMP-2	Preclinical	Gaucher disease [57]

## Data Availability

Not applicable.
